# Analysis of the fecal microbiome and metabolome in dairy cows with different body condition scores

**DOI:** 10.1371/journal.pone.0319461

**Published:** 2025-03-10

**Authors:** Bhuripit Saraphol, Woranich Hinthong, Peerut Chienwichai, Natapol Pumipuntu, Onrapak Reamtong, Thassanee Srisook, Jiraphan Premsuriya

**Affiliations:** 1 Faculty of Veterinary Sciences, Mahasarakham University, Maha Sarakham, Thailand; 2 Princess Srisavangavadhana Faculty of Medicine, Chulabhorn Royal Academy, Bangkok, Thailand; 3 Research Center on Clinical and System Microbiology, Chulabhorn Royal Academy, Bangkok, Thailand; 4 One Health Research Unit, Mahasarakham University, Maha Sarakham, Thailand; 5 Department of Molecular Tropical Medicine and Genetics, Faculty of Tropical Medicine, Mahidol University, Bangkok, Thailand; Central University of Andhra Pradesh, INDIA

## Abstract

Holstein Friesian is the most popular breed of dairy cows worldwide due to its exceptional milk production capabilities. In dairy cow management, the body condition score (BCS) is a useful tool, serving as a reliable indicator of a cow’s nutritional status and overall health. It is determined via a subjective visual and tactile assessment of fat cover and muscle mass. A low BCS is associated with decreased milk production and fertility. While genetic and nutritional factors have previously been associated with BCS, their effects are often moderate. In this study, we compared the fecal microbiome and the untargeted fecal metabolome of normal (BCS ≥  3, n =  16) and thin (BCS <  3, n =  16) Holstein Friesian dairy cows. The 16S rRNA gene-based metagenomic analysis revealed that thin cows had significantly higher levels of Clostridiaceae, Erysipelotrichales, Erysipelotrichaceae, and *Turicibacter*, while normal cows had higher levels of Clostridiales_vadinBB60_group, UCG-010, Bacteroidaceae, Ruminococcaceae, Paludibacteraceae, *Alistipes*, and *Bacteroides*. The fecal metabolomic analysis showed that key signaling pathways, including the mechanistic target of rapamycin (mTOR), phosphatidylinositol 3-kinase (PI3K)-Akt, and AMP-activated protein kinase (AMPK) pathways, were enriched in thin cows. In addition, a significant correlation was observed between differential microbial taxa and metabolites. Notably, Clostridiaceae and Erysipelotrichaceae species are linked to inflammation, infectious diseases, and conditions such as ruminal acidosis. Additionally, the mTOR, PI3K-Akt, and AMPK pathways are known to be activated by both nutrient deficiencies and inflammation. We propose that, in addition to genetic and nutritional factors, gut microbiome dysbiosis may contribute to subclinical health conditions, such as chronic inflammation and acidosis, which indirectly affect the cow’s BCS. These findings are guiding our ongoing research on the underlying health conditions in thin cows to better understand the role that the gut microbiome plays in the regulation of the body condition.

## Introduction

Holstein Friesian is the most widely recognized and popular breed of dairy cattle in the world. This breed plays a pivotal role in the global dairy industry due to its exceptional milk production capabilities. With careful management and attention to health and nutrition, Holstein Friesian cattle can thrive and contribute significantly to dairy operations [1,2]. Established breeding programs continue to enhance the traits of this breed, making it a cornerstone of modern dairy farming [3–5].

The body condition score (BCS) is a critical tool used in dairy cattle management. It is a reliable indicator of a cow’s nutritional status and overall well-being. The BCS is determined by performing a subjective visual and tactile evaluation of the fat cover and muscle mass on a cow’s body, and it is typically quantified using a scale of 1 to 5 (1 =  extremely thin, 5 =  excessively fat) [6–8]. Maintaining an optimal BCS is essential for maximizing both production and reproductive performance; a low BCS can lead to reduced milk production and fertility, while an excessively high BCS can increase the risk of metabolic disorders [6,7]. By effectively managing each cow’s BCS throughout the lactation cycle, producers can enhance their herd’s overall efficiency and profitability [6,9,10]. Various factors influence or are associated with the BCS in dairy cows. Several studies have demonstrated moderate heritability of BCS values in Holstein Friesian cows; however, genetic correlation estimates differ across studies [11–14]. Management practices and nutrition also play significant roles in determining a cow’s BCS [8,15,16].

The microbiome and metabolome of dairy cows have gained significant attention in recent years due to their potential effects on animal health, nutrition, and productivity [17–19]. The microbiome and metabolome are closely interconnected. Microorganisms produce a variety of metabolites that can influence the host’s physiology and metabolism [17–19]. In turn, the host’s diet, genetics, and health status can impact the composition and function of the microbiome [18,20,21]. To date, limited comparative studies have been conducted on the microbiome and metabolome of dairy cows with different BCS values. In a study that examined the relationship between the rumen microbiome and metabolome and the BCS in prepartum Holstein Friesian cows, it was found that certain microbial taxa were significantly more abundant in cows with a high BCS compared to those with a low BCS [22]. It was also found that the citrate cycle was significantly enriched, with a notable increase in citrate levels, and this increase was strongly linked to the bacterial genera that were more abundant in cows with high BCS values.

The presence of a balanced and effective rumen microbiome is essential for the health and productivity of dairy cows. By breaking down plant material and producing nutrients, these microbial communities contribute significantly to the host’s BCS [23]. However, the gut microbiome can also influence dairy cow health beyond nutrient consumption. Imbalances in the gut microbiome have been linked to various diseases, including intestinal ulcers, metritis, diarrhea, reticuloperitonitis, and ruminal acidosis, which can negatively impact a cow’s health and overall well-being [19,24].

In this study, we aimed to investigate the fecal microbiome and metabolome of Holstein Friesian cows with normal and low BCSs using 16S rRNA gene sequencing and an untargeted metabolomic approach. By doing so, we sought to extend the analysis of the factors that influence the BCS and thus obtain a deeper understanding and broader perspective of the contributing factors in this breed.

## Materials and methods

### Sample collection

**Fig 1:**
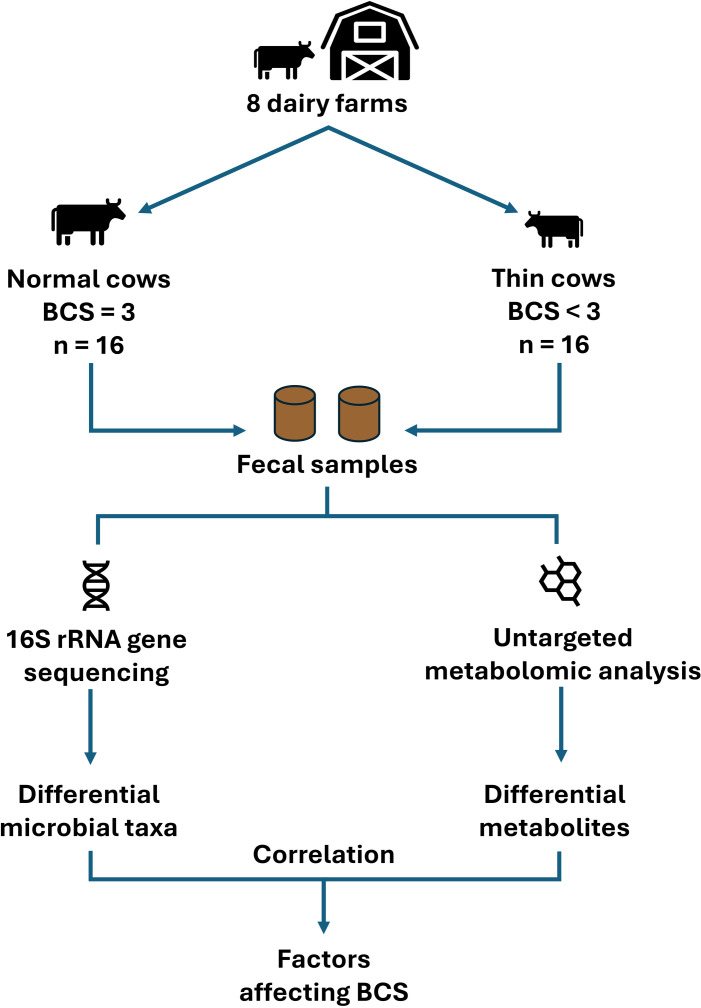
Schematic diagram of the experimental workflow.

A total of 32 fecal samples were collected from Holstein Friesian cows across 8 dairy farms in Maha Sarakham province, Thailand. The fecal samples were divided into two groups: those from cows with a low BCS ( < 3.0; n =  16; the thin group), and those from cows with a normal BCS (3.0; n =  16; the normal group). All farms involved in this study were members of a cooperative and followed similar feeding practices. For heifers, the feed consisted of a roughage-to-concentrate ratio of 60:40. The roughages included cassava peels, corn silage, grass, and straw from local areas, while the concentrates were supplied by the cooperative and contained more than 16% protein. All participating farms were certified with Good Agricultural Practices for Dairy Cattle Farms by the National Bureau of Agricultural Commodity and Food Standards, ensuring compliance with uniform feeding and management guidelines. All cows included in this study were non-pregnant heifers aged over one year. The numbers of normal and thin cows from each farm are provided in S1 Table. The sample collection was approved by the Institutional Animal Care and Use Committee of Mahasarakham University (license number: IACUC-MSU-27/2023). The fecal samples were collected in sterile bags by a veterinarian. All samples were stored in an ice box immediately after collection, transferred to the laboratory, and stored at − 80^o^C until further experimentation. The experimental design is illustrated in Fig 1.

### Extraction of DNA from fecal samples

The total DNA of the fecal samples was extracted using a QIAamp PowerFecal Pro DNA Kit (Qiagen, Germany) according to the manufacturer’s instructions. Briefly, each fecal sample was added to a bead beating tube. Buffer was then added, and the tube was vortexed to lyse the cells. DNA was captured using a silica membrane spin column and then washed and eluted. The DNA concentration and purity were measured using a Nanodrop (Thermo Scientific, USA). Samples with a concentration >  50 ng/μL and an OD 260/280 ratio of 1.8–2.0 were selected for sequencing. All DNA samples were stored at − 80^o^C until sequencing.

### Sequencing and bioinformatics analysis

The sequencing of the 16S rRNA gene and bioinformatics analysis were performed using the Illumina Hiseq 2500 platform (Novogene, China). The DNA samples were used as templates for the amplification of the V3–V4 hypervariable region of the 16S rRNA gene by polymerase chain reaction (PCR) using the barcoded 16S rRNA universal primer, 341F primer 5’-CCTAYGGGRBGCASCAG-3’ and 806R primer 5’-GGACTACNNGGGTATCTAAT-3’. The PCR product was ligated with an Illumina adapter, and the libraries were sequenced. The paired-end reads from the sequencing were merged using FLASH (V1.2.11, http://ccb.jhu.edu/software/FLASH/) [25], and fastp software (version 0.20.0) [26] was used to filter the raw tags and obtain clean tags, which were then compared with the references in the Silva database (version 138.1, https://www.arb-silva.de/) [27]. VSEARCH (version 2.15.09) [28] was used to detect and remove chimeric sequences.

Amplicon sequence variants (ASVs) were denoised using DADA2 in QIIME2 (version QIIME2-202005) to obtain the initial ASVs [29], and ASVs with an abundance value <  5 were discarded from the analysis. Species annotation was performed using QIIME2 with the Silva database. Two-sample t-tests and Wilcoxon rank-sum tests were conducted using R software (version 2.15.3) to compare the relative abundances of individual taxa in normal and thin cows, with statistical significance determined at a *p*-value of 0.05.

### Analysis of species richness and diversity

Alpha and beta diversities were calculated using QIIME2. The alpha diversity results were used to determine the bacterial community in each group, with reference to the Shannon and Simpson indices, and the taxonomic richness was indicated by the Chao1 value. Beta diversity was visualized by nonmetric multidimensional scaling (NMDS) based on the Bray–Curtis dissimilarity of the ASV composition. The differences in the bacterial community among the samples from each cow group were calculated by conducting an analysis of similarity using ADONIS functions (*p* <  0.05) in QIIME2.

### Extraction of metabolites from fecal samples

Metabolites were extracted from the fecal samples using a previously described method, with some modifications [30]. Briefly, approximately 100 mg of feces was mixed with ice-cold isopropanol at a 1:4 ratio (w/v). To ensure the complete dissolution of metabolites, the mixture was vigorously shaken for 2 min and sonicated on ice for 5 min. The supernatant was collected after centrifugation (14,700 g, 15 min at 4°C), transferred to a new tube, and dried using a speed vacuum machine (Tomy Digital Biology, Tokyo, Japan).

### Metabolite identification

Metabolites were identified using an ultra-high performance liquid chromatography (UHPLC) instrument (Agilent 1260 Quaternary Pump, Agilent 1260 High Performance Autosampler, and Agilent 1290 Thermostatted Column Compartment SL, Agilent Technologies, USA) coupled to a quadrupole time-of-flight mass spectrometry (QTOF-MS) instrument (TripleTOF 5600 + , SCIEX, USA) with DuoSpray ion source electrospray ionization (ESI). For the chromatographic separation, a 1:1 (v/v) mixture of 0.1% formic acid in water (mobile phase A) and 0.1% formic acid in acetonitrile (mobile phase B) was used to resuspend the dried metabolite samples. The samples were kept at 6˚C and automatically injected into the UHPLC system, which was equipped with a C18 reversed-phase column (ACQUITY UPLC BEH, 2.1 ×  100 mm, 1.7 µ M, Waters, USA) and operated at a flow rate of 0.3 mL/min at 40°C. Analyst Software (version 1.7, SCIEX) was used to acquire the mass ion chromatograms and mass spectra from the QTOF-MS instrument in both the positive (+ESI) and negative (−ESI) ESI modes. An information-dependent data acquisition process was used to acquire a TOF-MS scan and 10 dependent product ion scans using the high sensitivity mode with dynamic background subtraction. The mass range of the TOF-MS scan was *m/z* 100–1,000, and the product ion scan was set to *m/z* 50 − 1,000. Quality control (QC) samples, which were created by pooling equal aliquots of each metabolite sample, were injected before, during (after every fourth sample), and after sample analysis to evaluate the system’s performance.

### Metabolite annotation

Metabolites were annotated using the XCMS online platform (version 3.7.1, https://xcmsonline.scripps.edu/landing_page.php?pgcontent=mainPage) [31]. The raw.wiff and.wiff.scan files were uploaded to the XCMS server and later analyzed in a pairwise manner. Regarding feature extraction, the polarity was selected as either positive or negative, depending on the type of data. The parameters for maximal tolerated *m/z* deviation, second peak width, signal/noise threshold, and minimum difference in *m/z* were set to 15, 5–20, 6, and 0.01, respectively. For the alignment processes, the allowable retention time duration was set to 5 s, with a 0.5 minimum fraction and a 0.015 width of overlapping *m/z*. The annotation parameters included a 5 ppm error, a 0.01 *m/z* absolute error, and an isotopic search for the features and their adduct formations. Regarding the identification processes, the METLIN database [32]was searched, considering common adducts with a 5-ppm error tolerance. The raw metabolomic data are shown in S2 Table.

### Metabolomic data preprocessing and analysis

The *m/z* and peak intensity data of each metabolomic feature were exported from the XCMS platform and statistically analyzed using the MetaboAnalyst online platform (version 5.0, https://www.metaboanalyst.ca/) [33]. Using the Statistical Analysis [one factor] module, the median intensity of each peak was preprocessed with interquartile range filtration, quantile normalization, cube root transformation, and range scaling. In addition, the principal component analysis (PCA) algorithm was used to evaluate the reliability of the metabolite identification system by analyzing all the metabolomic data together with the data from the QC samples. The acceptance criterion for high-quality data was that the QC samples should be clustered in the middle of the PCA plot [34]. Datasets that met this criterion were further analyzed. The metabolomic data were visualized using a partial least squares discriminant analysis (PLS-DA) score plot, a hierarchical clustering heatmap, and a volcano plot. In the multivariate PLS-DA scores plot, the 95% confidence regions are displayed to enable the evaluation of the separation of the metabolomic data across the groups. To further investigate the metabolite patterns in normal and thin cows, a hierarchical clustering heatmap showing the top 50 metabolites was generated with the Euclidean distance measure and Ward clustering. Moreover, a volcano plot was generated to pinpoint the significantly different features (i.e., those with a t-test *p*-value <  0.05 and fold change value ≥  2).

### Pathway analysis

A pathway analysis of the significantly different metabolites was performed using the STITCH platform (version 5.0, http://stitch.embl.de/) [35]. All putative metabolites with a *p*-value <  0.05 and a fold change ≥  2 were added to the server with the “*Bos taurus*” organism criteria. The condition “medium confidence (0.400)” was selected for the minimum required interaction score, and no more than 10 interactions were shown for the first and second shells.

### Correlation analysis

Spearman’s rank correlation test was performed using PAST software [36] to assess the correlation between significantly different microbial taxa and metabolites. Results were considered significant when the *p-*value was <  0.05.

## Results

### The fecal microbiomes of normal and thin cows differed

**Fig 2:**
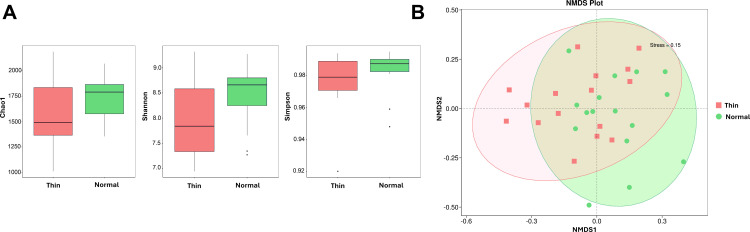
Diversity of the fecal microbiome in normal and thin cows according to 16S rRNA gene sequencing data. **(A)** Box plots representing the alpha diversity indices (Chao1, Shannon, and Simpson indices). **(B)** Bray–Curtis nonmetric multidimensional scaling (NMDS) plot based on amplicon sequence variant (ASV) composition.

The 16S rRNA gene sequencing generated 6,477,917 raw reads, and there was an average of 202,435 reads per sample. After QC and filtering, 12,031 ASVs were identified. The alpha diversity analysis conducted at the ASV level revealed no significant differences between the samples from normal (BCS ≥  3) and thin (BCS <  3) Holstein Friesian cows in terms of the Chao1, Shannon, and Simpson indices *(p* >  0.05) (Fig 2A). However, the beta diversity analysis based on the Bray–Curtis dissimilarity indicated significant differences in the fecal microbiota structure between the two groups (Fig 2B). All sequenced reads generated during this study have been deposited in the Sequence Read Archive (SRA) of the National Center for Biotechnology Information (NCBI) under accession number PRJNA1173238.

**Fig 3:**
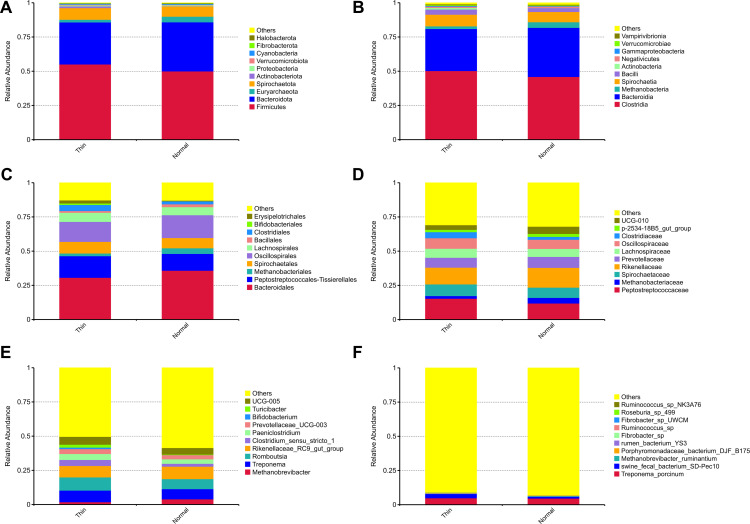
Stacked bar plots illustrating the relative abundance distribution of fecal microbial communities in thin and normal cows. **(A)** Top 10 most abundant phyla. **(B)** Top 10 most abundant orders. **(C)** Top 10 most abundant families. **(D)** Top 30 most abundant genera.

This was confirmed by ADONIS analysis, which revealed a significant difference in the fecal microbiota structure of dairy cows with different BCS values (*p* =  0.035). The predominant phyla in the fecal microbiota of both normal and thin cows were Firmicutes, Bacteroidota, Euryarchaeota, and Spirochaetota (Fig 3A). The overall taxonomic composition at the order, family, and genus levels differed between the two groups (Figs 3B and 3D). The two-sample t-tests or Wilcoxon rank-sum tests revealed significant differences in the abundance of certain taxa between normal and thin cows (*p* <  0.05).

**Fig 4:**
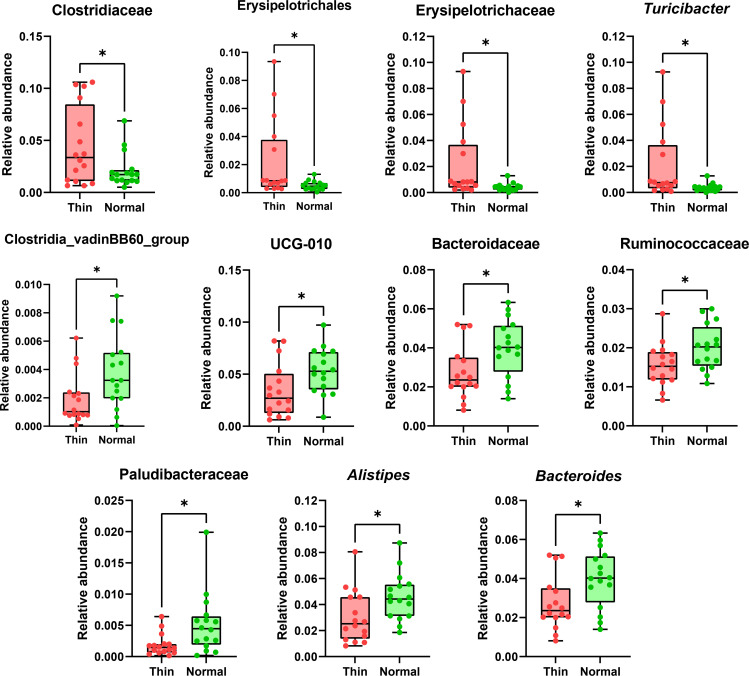
Box plots showing the taxa that were significantly different between thin and normal cows. Asterisks (*) indicate significant differences based on t-test or Wilcoxon rank-sum tests results (*p* <  0.05).

Thin cows had significantly higher levels of Clostridiaceae, Erysipelotrichales, Erysipelotrichaceae, and *Turicibacter* compared to normal cows. Conversely, normal cows had significantly higher levels of Clostridiales_vadinBB60_group, UCG-010, Bacteroidaceae, Ruminococcaceae, Paludibacteraceae, *Alistipes*, and *Bacteroides* (Fig 4). Taxa that had a relative abundance of less than 0.1% were considered “low abundance” and were not included in the analysis.

### The fecal metabolites of normal and thin cows differed

The metabolomic data were subjected to a quality assurance process to ensure the reliability of the MS-based identification of the metabolites. The predefined criterion for this quality assurance was that the QC samples had to cluster together in the PCA plot. As shown in S1 Fig, the QC samples were located close to each other in the middle-left area of the PCA plot. This finding indicated that the metabolite identification procedure was reliable and consistent. After confirming the quality of the procedure, we performed the data analysis.

**Fig 5:**
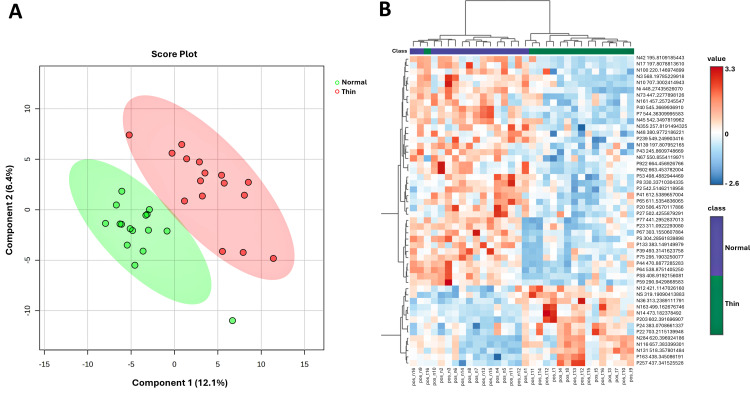
Multivariate and clustering analysis of metabolomic data. **(A)** Partial least squares discriminant analysis (PLS-DA) score plot showing the separation of the samples from the normal (green dots) and thin (red dots) cows. The ellipses represent the 95% confidence regions. **(B)** Hierarchical clustering heatmap of the top 50 metabolites. Red indicates features with increased intensity, and blue indicates features with decreased intensity. The purple and green bars indicate the data from normal and thin cows, respectively.

The PLS-DA score plot showed that fecal metabolites in the samples from the normal group were distinct from those in the samples from the thin group (Fig 5A). This result suggested that the fecal metabolites of the two groups of cows were different. We also generated a hierarchical clustering heatmap of the top 50 metabolites to explore and compare the metabolite profiles of the individual cows in the two groups (Fig 5B). The results showed that there were similarities within the groups and differences between the groups.

**Fig 6:**
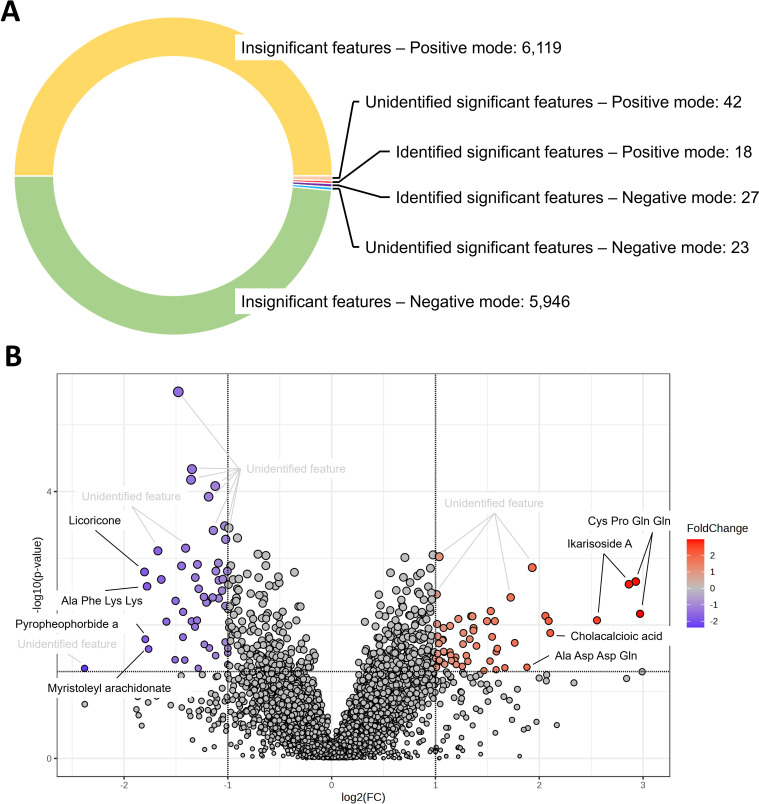
Analysis of the significantly different metabolomic features. **(A)** The number of metabolomic features identified in the metabolomic analysis of the cow fecal samples. **(B)** Volcano plot highlighting the significantly different metabolomic features. The red dots represent the features with significantly increased intensity, and the purple dots represent the features with significantly decreased intensity. The dashed lines indicate the fold change and *p*-value cutoffs.

Of the 12,175 features identified using both positive and negative modes, 110 features were considered to be significantly different. Approximately 41% (n =  45) of the features were assigned as putative metabolites (Fig 6A). We then generated a volcano plot to screen for metabolites present at significantly different levels in the two groups. Despite the fact that many features could not be matched to existing compounds in the METLIN database, several interesting putative metabolites were identified, for example, cholacalcioic acid and ikarisoside A (Fig 6B).

The top 10 putative metabolites found to be at higher levels in the fecal samples from thin cows compared to those from normal cows are shown in Table 1. Similarly, the top 10 putative metabolites found to be at lower levels in the fecal samples from thin cows compared to those from normal cows are also shown in Table 2.

**Table 1:** The top 10 putative metabolites with the largest positive fold change between normal and thin cows.

No.	Median *m/z*	Ionization mode	Putative metabolite	METLIN ID	Ion adducts	Mass error (ppm)	*p*-value	Fold change
1	473.1822	Negative	His Met Ser Thr	155795	M-H	0	0.0068	7.839
2	473.1823	Negative	Cys Pro Gln Gln	116553	M-H	0	0.0022	7.617
3	499.1626	Negative	Phellamurin	67946	M-H_2_O-H	4	0.0025	7.281
4	499.1625	Negative	Ikarisoside A	50126	M-H	3	0.0085	5.881
5	371.2596	Negative	Cholacalcioic acid	41962	M-H	1	0.0132	4.305
6	417.2643	Negative	Simvastatin	2443	M-H	1	0.0087	4.259
7	373.2739	Positive	3-Ethoxyandrosta-3,5-dien-17beta-ol propanoate	70551	M + H	1	0.0073	4.167
8	428.1417	Negative	Ala Asp Asp Gln	104333	M-H_2_O-H	0	0.0429	3.684
9	645.4694	Positive	Rolliniastatin-1	46760	M + Na	1	0.0185	3.396
10	303.2179	Negative	2,15,16-Trihydroxy palmitic acid	35476	M-H	1	0.0438	3.179

**Table 2:** The top 10 putative metabolites with the largest negative fold change between normal and thin cows.

No.	Median *m/z*	Ionization mode	Putative metabolite	METLIN ID	Ion adducts	Mass error (ppm)	*p-*value	Fold change
1	383.1491	Positive	Licoricone	47589	M + H	1	0.0016	-3.4894
2	535.2719	Positive	His His Lys Asn	154050	M + H	3	0.0163	-3.4713
3	493.3141	Positive	Ala Phe Lys Lys	105248	M + H	2	0.0026	-3.4307
4	521.4313	Positive	Myristoleyl arachidonate	97105	M + Na	3	0.0229	-3.3954
5	568.1978	Negative	Asp His Met Trp	122098	M-H_2_O-H	0	0.0021	-3.1193
6	535.2704	Positive	Pyropheophorbide a	63917	M + H	0	0.0152	-2.6991
7	423.2360	Positive	Ala His Pro Val	106137	M + H	2	0.0088	-2.5471
8	303.1550	Positive	Arginyl-glutamate	85623	M + H	4	0.0019	-2.4859
9	716.4830	Positive	PS(O-16:0/14:0)	78650	M + Na	1	0.0038	-2.3464
10	536.2739	Positive	Glu Phe Ile Gln	129233	M + H	5	0.0195	-2.3403

### Signaling pathways were perturbed in thin cows

**Fig 7:**
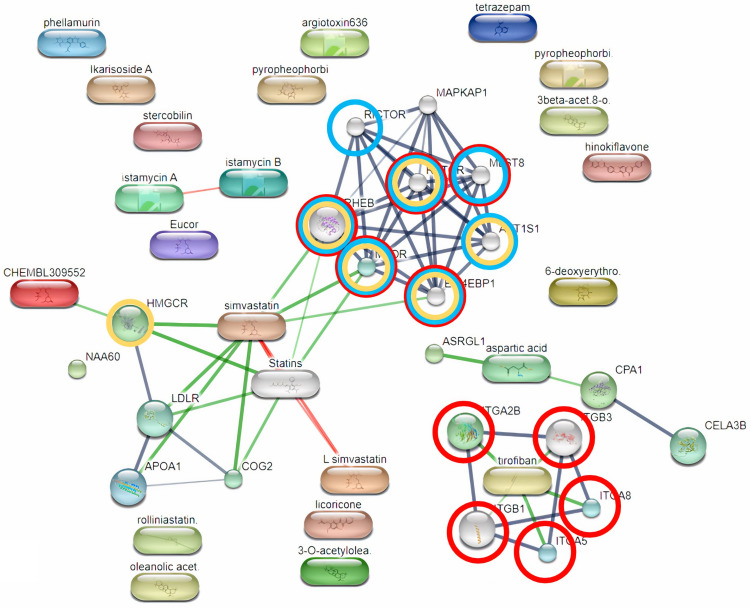
Results of the pathway analysis performed using the STITCH bioinformatics tool. The blue, red, and yellow circles show nodes in the mechanistic target of rapamycin (mTOR), phosphatidylinositol 3-kinase (PI3K)-Akt, and AMP-activated protein kinase (AMPK) signaling pathways, respectively.

The significantly different putative metabolites were analyzed further to identify the pathways that were affected in thin cows. Using the STITCH platform, we determined that the identified compounds were enriched in 15 pathways from the Kyoto Encyclopedia of Genes and Genomes (KEGG) database [37] (S3 Table). The protein–protein enrichment *p*-value was 0.000463, indicating the significance of the pathway analysis. Among the enriched pathways, the top three were signaling pathways: the mechanistic target of the rapamycin (mTOR) signaling pathway, the phosphatidylinositol 3-kinase (PI3K)-Akt signaling pathway, and the AMP-activated protein kinase (AMPK) signaling pathway (Fig 7).

### Correlation between the fecal microbiota and metabolites

**Fig 8:**
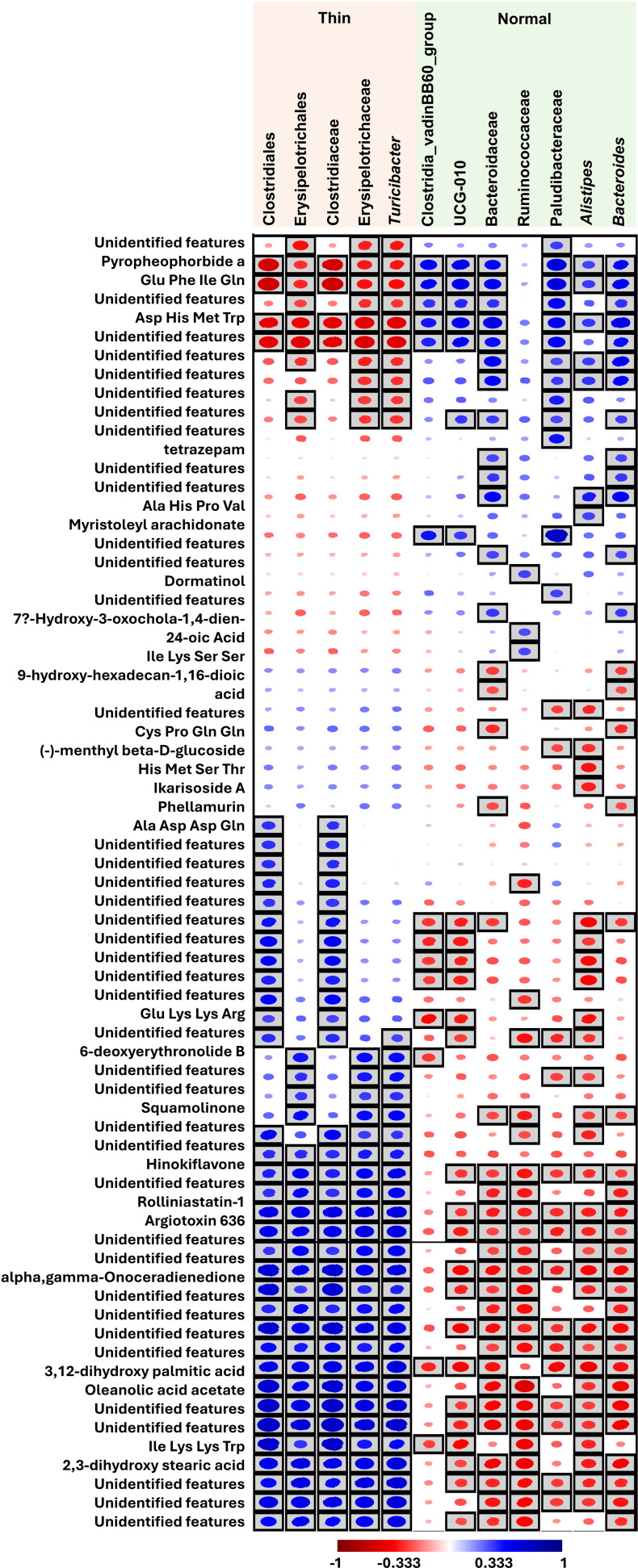
Correlations between significantly different microbiota and metabolites. The rectangular boxes indicate significance (*p* <  0.05). Blue and red represent positive and negative correlations, respectively.

Spearman’s rank correlation test was used to assess the correlation between significantly different microbial taxa and metabolites. A substantial portion (64.5%, 71/110) of the fecal metabolites exhibited significant correlations with the fecal microbial taxa (*p* <  0.05) (Fig 8). Notably, taxa that were significantly more abundant in thin cows compared to normal cows exhibited inverse correlations with many metabolites. However, the majority of the significantly correlated metabolites remain unidentified, which limited our ability to reconstruct the metabolic networks or pathways from the data.

## Discussion

Holstein Friesians are widely recognized for their exceptional milk production, underscoring their importance in global dairy farming [1,2]. The BCS is a key tool for assessing the nutritional status and well-being of dairy cows and ensuring optimal animal health and productivity [6,7]. This study uniquely contributes to understanding BCS variations in Holstein Friesians by highlighting the role of the gut microbiome and metabolome. By identifying significant differences in microbial taxa and metabolic pathways between cows with normal and low BCS, this research provides evidence that microbial dysbiosis and altered metabolic signaling pathways are critical factors influencing BCS. These findings deepen our understanding of the biological processes underlying BCS variations and provide new insights for addressing health challenges in dairy herds. In this study, we analyzed the fecal microbiome and metabolome of normal (BCS =  3) and thin (BCS <  3) Holstein Friesian dairy cows. Alpha diversity which reflects species richness and diversity within each group and beta diversity which assesses differences in community composition between groups were used to evaluate the bacterial community diversity in normal and thin cows. While no significant differences were observed in the alpha diversity, the beta diversity analysis revealed that the microbial community structure significantly differed between the two groups. Compared to normal cows, thin cows had significantly higher levels of Clostridiaceae, Erysipelotrichales, Erysipelotrichaceae, and *Turicibacter*.

Previous studies have shown that various Clostridiaceae species can cause gastrointestinal diseases in cattle, including diarrhea, enteritis, abomasitis, and hemorrhagic bowel syndrome [38–40]. Furthermore, Clostridiaceae species were found to be more abundant in the rumen of dairy cows with lower milk protein yields and negatively associated with milk protein content [41]. Addressing these microbial imbalances may involve targeted interventions, such as dietary adjustments to promote a healthy gut microbiome or the use of probiotics to reduce the abundance of pathogenic species. For example, incorporating high-fiber feed or specific prebiotics could help create a favorable environment for beneficial microbes, potentially mitigating the negative impacts of Clostridiaceae overgrowth [42–44].

Within the Firmicutes phylum, members of the Erysipelotrichaceae family are commonly found in the cattle digestive tract, and dietary variations have been shown to have a significant impact on the abundance of Erysipelotrichaceae species within the cow’s gut microbiome [45–47]. Most Erysipelotrichaceae species can ferment a diverse array of sugars to produce lactic acid [46,48]. Notably, an increased abundance of Erysipelotrichaceae species has been linked to a higher risk of ruminal acidosis in dairy cows [49], which is caused by substantial increases in lactic acid in the rumen. It is not classified as a distinct disease but rather as a spectrum of ruminal acidity levels. A drop in pH below the normal range significantly affects microbial activity, rumen function, and overall animal productivity and health [50]. This aligns with the observation that thin cows may have experienced dietary imbalances, potentially including high levels of readily fermentable carbohydrates, which could exacerbate the growth of these bacteria and contribute to ruminal acidosis [51,52].

*Turicibacter* species (phylum Bacillota) reside in the digestive tract of many mammals, including cattle [53,54]. However, the role that *Turicibacter* species play in cattle remains uncharacterized. A previous study demonstrated the ability of *Turicibacter* strains to modify bile acids and host lipids; mice inoculated with *Turicibacter* strains exhibited decreased levels of serum cholesterol, triglycerides, and adipose tissue mass [54]. Therefore, the relatively higher levels of *Turicibacter* species observed in thin dairy cows in this study may have contributed to altered lipid metabolism, potentially influencing their lower BCS. Furthermore, a previous study identified a correlation between *Turicibacter* species and increased somatic cell count (SCC) in milk, which serves as a marker of mammary gland inflammation and subclinical mastitis in dairy cows [53,55]. These findings suggest a possible link between *Turicibacter* abundance and systemic metabolic and inflammatory processes in thin cows, warranting further research to clarify its role and potential impact on dairy cow health [53,55].

Normal cows demonstrated significantly higher levels of Clostridiales_vadinBB60_group, UCG-010, Bacteroidaceae, Ruminococcaceae, Paludibacteraceae, *Alistipes*, and *Bacteroides* compared to thin cows. Microbial taxa such as Clostridiales_vadinBB60_group and Ruminococcaceae have been associated with anti-inflammatory effects and healthy metabolic states in various studies. For example, Clostridiales_vadinBB60_group species, though their role in cattle remains unclear, tend to be more abundant in healthy control groups across different species. In mice, higher levels of these bacteria have been linked to reduced obesity, dyslipidemia, and insulin resistance [56]. Similarly, in broilers, these bacteria have been associated with decreased inflammation [57]. In humans, increased levels of Clostridiales_vadinBB60_group have been correlated with lower incidences of coronary artery disease and primary sclerosing cholangitis [56,58].

Other taxa, such as UCG-010, Bacteroidaceae, and Paludibacteraceae, are commonly found in the cattle gut microbiome and are influenced by dietary factors and animal age. These microbes are thought to contribute to healthy digestion and nutrient metabolism [59–65]. By grouping these microbial taxa based on their potential functional roles, this highlights their relevance to anti-inflammatory effects and healthy metabolic states, offering insight into their possible contribution to the better body condition observed in normal cows. Additionally, *Alistipes* species, which belong to the Rikenellaceae family, have been identified in the fecal microbiota of Holstein cows [66,67]. However, the specific role these species play in dairy cows remains unknown.

Our metabolomic analysis of the fecal samples revealed that the mTOR, PI3K-Akt, and AMPK signaling pathways were enriched in thin cows. The nutrient-sensing mTOR signaling pathway is a conserved mammalian pathway that plays a crucial role in the regulation of various cellular processes, including cell growth, proliferation, and metabolism. It acts as a central hub in a complex signaling network, integrating signals from various sources, such as nutrients, hormones, growth factors, and stress [68]. Previous studies conducted on dairy cows have also highlighted that the mTOR signaling pathway is involved in the modulation of the immune response [69,70], suggesting that this pathway may contribute to nutrient deficiencies and inflammation observed in thin cows. Modulating the mTOR pathway could potentially serve as a biomarker or therapeutic target to enhance nutritional status and immune function, thereby contributing to improved body condition [71,70].

The PI3K-Akt pathway is a crucial signaling pathway that regulates essential cellular functions, including gene expression, protein synthesis, and cell growth and survival. It is activated by various cellular signals and stressors [72]. Notably, research conducted with dairy cows has shown that viral and bacterial infections can trigger the activation of the PI3K/Akt/mTOR pathway [73,74]. Targeting this pathway might provide opportunities to manage infections or inflammation that indirectly affect BCS.

AMPK is a key energy sensor responsible for maintaining cellular homeostasis in eukaryotes. When activated, AMPK stimulates ATP synthesis while suppressing ATP-consuming processes, helping maintain the cellular energy balance. Nutrient deficits and various physiological stressors can elevate AMP/ADP levels, leading to AMPK activation [75]. In dairy cows, AMPK activation results in the oxidative degradation of lipids and the inhibition of fatty acid synthesis [76,77]. Furthermore, the AMPK signaling pathway is involved in alleviating inflammation in dairy cows [78]. Given its role in energy regulation and anti-inflammatory effects, AMPK activation could be explored as a biomarker for identifying cows at risk of poor body condition or as a target for nutritional or pharmaceutical interventions aimed at improving BCS [78,79].

Collectively, the signaling pathways found to be enriched in thin cows play crucial roles in cellular physiological processes and functions and are activated by nutrient deficiencies, which potentially stem from nutrient digestion and absorption issues. These findings suggest that a cow’s BCS may be closely linked to the gut microbial and metabolomic profiles. Notably, all the enriched pathways in thin cows, including mTOR, PI3K-Akt, and AMPK, are associated with immune responses and inflammation. This aligns with the observed higher levels of bacterial taxa in thin cows, such as Clostridiaceae and Erysipelotrichaceae, which are known to trigger inflammatory processes and contribute to conditions like ruminal acidosis. The connection between these microbial taxa and pathways highlights the potential role of inflammatory markers, such as interleukins and cytokines, in mediating the observed physiological changes in thin cows. Future studies could further elucidate this relationship by directly measuring these markers to validate the links between microbial imbalances, pathway activation, and systemic inflammation.

We hypothesize that dysbiosis of the cow’s gut microbiome contributes to subclinical conditions, which indirectly affect BCS. Unlike traditional models of BCS determination that primarily focus on genetic predisposition and dietary intake [14–16], these findings highlight the novel integration of microbiome and metabolome data as critical components in understanding the underlying mechanisms regulating BCS. This approach provides a more comprehensive framework for addressing subclinical health issues and improving body condition in dairy cows. Future research should focus on comparing the BCS, fecal microbiome composition, fecal and serum metabolome, and inflammatory markers (e.g., interleukins and cytokines) of normal and thin cows. Longitudinal studies would be particularly effective in capturing temporal changes and causative relationships between microbiome composition, metabolomic shifts, and inflammatory markers. Additionally, intervention-based studies, such as dietary trials or microbiome transplants, could provide direct evidence of how targeted modifications influence BCS and underlying health conditions. The resultant knowledge will enhance our understanding of the gut microbiome’s role in the development and progression of underlying health conditions and in the regulation of the cow’s body condition.

## Conclusions

In this study, we investigated and compared the fecal microbiome and metabolome in normal (BCS =  3) and thin (BCS <  3) Holstein Friesian dairy cows. The results showed that thin cows had significantly higher levels of Clostridiaceae, Erysipelotrichales, Erysipelotrichaceae, and *Turicibacter*. Notably, Clostridiaceae and Erysipelotrichaceae have been linked to inflammation, infectious diseases, and other health conditions (e.g., acidosis). Our metabolomic analysis revealed that key signaling pathways, including the mTOR, PI3K-Akt, and AMPK signaling pathways, were enriched in thin cows. Activation of these pathways is often associated with nutrient deficiencies and inflammation. We propose that, in addition to genetic and nutritional factors, gut microbiome dysbiosis may contribute to subclinical health conditions that lead to lower BCS values. These findings are guiding our ongoing research on the underlying health conditions in thin cows to better understand the gut microbiome’s influence on the BCS.

## Supporting Information

S1 Table The numbers of normal and thin cows from each farm. (XLSX)

S2 Table Raw metabolomic data. (CSV)

S3 Table The pathway analysis of fecal metabolomic data using STITCH platform and KEGG database. (DOCX)

S1 Fig The principal component analysis (PCA) plot utilized in the quality assurance process. The quality control (QC) samples (purple dots) clustered tightly together, indicating the quality of the metabolite identification process. The green dots represent data from the normal group, and the red dots represent data from the thin group.
(TIF)
