# Supplementary material for: Analysis of the fecal microbiome and metabolome in dairy cows with different body condition scores
Source: PLoS One. 2025 Mar 10;20(3):e0319461. doi: 10.1371/journal.pone.0319461 (PMC11893135; doi:10.1371/journal.pone.0319461)
Supplement: S3 Table — (DOCX) [file pone.0319461.s003.docx]

| No. | Pathway description | Pathway ID | Count in gene set | False discovery rate |
| --- | --- | --- | --- | --- |
| 1. | mTOR signaling pathway | 4150 | 7 | 1.52E-08 |
| 2. | PI3K-Akt signaling pathway | 4151 | 10 | 2.49E-07 |
| 3. | AMPK signaling pathway | 4152 | 6 | 2.40E-05 |
| 4. | Arrhythmogenic right ventricular cardiomyopathy (ARVC) | 5412 | 5 | 2.53E-05 |
| 5. | Hypertrophic cardiomyopathy (HCM) | 5410 | 5 | 4.07E-05 |
| 6. | ECM-receptor interaction | 4512 | 5 | 4.23E-05 |
| 7. | Dilated cardiomyopathy | 5414 | 5 | 4.23E-05 |
| 8. | Focal adhesion | 4510 | 5 | 0.00243 |
| 9. | Regulation of actin cytoskeleton | 4810 | 5 | 0.00243 |
| 10. | Insulin signaling pathway | 4910 | 4 | 0.00598 |
| 11. | MicroRNAs in cancer | 5206 | 4 | 0.00598 |
| 12. | Hematopoietic cell lineage | 4640 | 3 | 0.0178 |
| 13. | Proteoglycans in cancer | 5205 | 4 | 0.0257 |
| 14. | Thyroid hormone signaling pathway | 4919 | 3 | 0.0369 |
| 15. | Platelet activation | 4611 | 3 | 0.0474 |

**S3 Table.** **The pathway analysis of fecal metabolomic data using STITCH platform and KEGG database.**
